# Diosmetin Delays In Vitro Aging of Porcine Oocytes by Improving Mitochondrial Function and Reducing Oxidative Stress

**DOI:** 10.3390/ani15030291

**Published:** 2025-01-21

**Authors:** Jia-Jun Ren, Xiu-Wen Yuan, Yu-Hao Zhang, Zi-Long Meng, Xing-Wei Liang, Nam-Hyung Kim, Yong-Nan Xu, Ying-Hua Li

**Affiliations:** 1 Guangdong Provincial Key Laboratory of Large Animal Models for Biomedicine, South China Institute of Large Animal Models for Biomedicine, School of Pharmacy and Food Engineering, Wuyi University, Jiangmen 529000, China; r2075611233@163.com (J.-J.R.); yeahwwen66@163.com (X.-W.Y.); 15271723044@163.com (Y.-H.Z.); 18119510223@163.com (Z.-L.M.); nkkim@wyu.edu.cn (N.-H.K.); ynxu0613@wyu.edu.cn (Y.-N.X.); 2 College of Animal Science & Technology, Guangxi University, Nanning 530004, China; xwliang@gxu.edu.cn

**Keywords:** diosmetin, autophagy, apoptosis, mitochondrial, oocyte aging

## Abstract

Oocyte quality is crucial for successful fertilization and subsequent embryonic development. Post-ovulatory aging leads to reduced oocyte quality and impaired embryogenesis, representing an unavoidable challenge in terms of certain assisted reproductive techniques. Diosmetin (DIOS), a natural flavonoid found in lemons, spearmint, and spider moss, exhibits antioxidant, anti-inflammatory, and anti-apoptotic properties. However, its effects on the aging of mature porcine oocytes in vitro remain unexplored. This study investigated the impact of DIOS on porcine oocyte aging. In the IVM medium, fresh oocytes were cultured for 44 h, while aging oocytes were cultured for 68 h. Following the addition of varying DIOS concentrations (0.01, 0.1, and 1 μM) to the IVM medium, the DIOS-treated aging oocyte group was cultured for 68 h. The results demonstrated that 0.1 μM DIOS significantly improved the blastocyst rates and cell counts, reduced the reactive oxygen species (ROS), elevated the glutathione (GSH) levels, enhanced the mitochondrial function, and decreased the markers of autophagy (LC3B), apoptosis (annexin V), endoplasmic reticulum stress (CHOP), and senescence (SA-β-Gal). Furthermore, DIOS treatment upregulated the expression of relevant genes compared to the aged group. These findings suggest that DIOS effectively delays porcine oocyte aging.

## Simple Summary

Diosmetin, a potent antioxidant, significantly mitigates oxidative stress and enhances mitochondrial function. When incorporated into the in vitro maturation medium, it was found to improve the quality of aging oocytes and promote subsequent embryo development.

## 1. Introduction

During oocyte maturation, whether in vivo or in vitro, oocytes in the metaphase II (MII) stage of the second meiotic division undergo progressive degeneration if not fertilized promptly [1]. This phenomenon, termed post-maturation oocyte aging (PMOA), occurs in vivo when oocytes remain in the fallopian tubes after ovulation, as fertilization may not occur immediately following ovulation [2]. Similarly, oocytes cultured to maturity for micromanipulation and in vitro fertilization often experience extended maturation periods, leading to inevitable aging due to individual variations in the maturation timing [3].

PMOA is characterized by various oocyte defects, including chromosomal structural anomalies and alterations in the zona pellucida, plasma membrane, cortical granules, mitochondria, and meiotic spindle [4]. This aging process is accompanied by biochemical and molecular changes, such as elevated reactive oxygen species (ROS) production, diminished maturation-promoting factor (MPF) activity, reduced expression of anti-apoptotic proteins like BCL-2, CASPASE3 activation, decreased glutathione (GSH) levels, and disrupted Ca^2+^ oscillatory signaling [5,6,7,8,9,10]. Aged oocytes, when exposed to sperm cytoplasmic factors to induce a fertilization-like Ca^2+^ response, frequently undergo cell death without completing activation [11].

Aged oocytes also exhibit extensive cytoplasmic and DNA damage, along with activation of CASPASE proteins [12]. Oocyte aging is closely associated with oxidative stress and mitochondrial dysfunction, both of which impair the oocyte quality and developmental potential [13]. Oxidative stress, primarily caused by an excessive accumulation of ROS generated by mitochondria, intensifies with aging [14,15], leading to compromised mitochondrial function [16]. Consequently, oocyte aging results in fertilization failure, poor or arrested embryonic development, higher miscarriage rates, and reduced offspring longevity [17,18]. Given these detrimental effects, strategies to delay oocyte aging are essential for promoting healthy embryo growth and development.

Oocyte aging is an irreversible process, although it can be delayed through external interventions. Advances in research have identified agents such as melatonin [19], ferulic acid [20], and apigenin [21] that mitigate oocyte aging, thereby improving the quality of aged oocytes. Diosmetin (DIOS), a natural flavonoid derived from lemon, exhibits both antioxidant [22] and anti-apoptotic properties [23] and has been shown to support the growth and development of early porcine embryos. However, its impact on oocyte aging remains unexplored. Notably, DIOS has been reported to ameliorate the mitochondrial dysfunction induced by a high-fat diet by upregulating the genes associated with mitochondrial biogenesis and dynamics, increasing the ATP levels, and reducing the oxidative stress in a high-fat-diet-induced obese rat model [24]. Given that oocyte aging is often linked to oxidative stress and mitochondrial dysfunction [13], it is hypothesized that DIOS may enhance mitochondrial function by alleviating oxidative stress, thereby delaying oocyte aging and improving oocyte quality.

This study investigated the effects of DIOS on oocyte aging, evaluating parameters such as the cleavage rate, blastocyst formation, cell count, antioxidant activity, mitochondrial function, senescence-associated β-galactosidase activity, endoplasmic reticulum stress (ERS), and levels of autophagy and apoptosis in porcine oocytes aged in vitro. The results will provide deeper insights into the molecular mechanisms regulating oocyte quality control, offering valuable perspectives for delaying oocyte aging and enhancing the reproductive potential of animals.

## 2. Materials and Methods

### 2.1. Animals and Chemicals

Unless otherwise specified, all the chemicals and reagents used in this study were purchased from Sigma-Aldrich (St. Louis, MO, USA). All the procedures were performed on a heating table maintained at 38.5 °C. The porcine ovaries were obtained from a slaughterhouse in Jiangmen, China.

### 2.2. Ethical Statement

The sow ovaries used in this study were obtained from sows that had already been slaughtered in local abattoirs, and there were no ethical issues involved.

### 2.3. Oocyte Collection and Maturation In Vitro

Ovaries from adolescent sows were collected from a local abattoir, placed in 0.9% saline containing penicillin and streptomycin, and transported to the laboratory within two hours. The ovaries were washed three times with saline, and cumulus–oocyte complexes (COCs) were aspirated from the follicles using an 18-gauge needle. The COCs were washed three times in TL-HEPES solution and five times in M199 culture medium before being transferred to 500 μL of in vitro maturation (IVM) medium (M199, Thermo Fisher Scientific, #11150-059, Waltham, MA, USA) supplemented with 0.91 mM sodium pyruvate, 10 IU/mL luteinizing hormone (Ningbo No. 2 Hormone Factory, Ningbo, China), 10 ng/mL epidermal growth factor, and 10 IU/mL follicle-stimulating hormone (Ningbo No. 2 Hormone Factory, Ningbo, China). The culture was covered with mineral oil (ART-4008P, SAGE, Suzhou City, China) in a four-well plate and incubated at 38.5 °C, 5% CO_2_, and 100% relative humidity. The fresh group underwent 44 h of IVM, while the aging group was cultured for 68 h. The aging treatment group was incubated in an IVM medium containing varying concentrations of DIOS (MCE, #HY-NO125) (0.01, 0.1, and 1 μM) for 68 h, without altering the medium composition. Mature MII oocytes were collected after 44 h for the fresh group and after 68 h for both the DIOS-treated and aging groups.

### 2.4. Parthenogenetic Activation (PA) and Embryo In Vitro Culture (IVC)

After 44 or 68 h of IVM, the COCs were treated with 0.1% hyaluronidase to remove the surrounding cells, and oocytes with extruded first polar bodies were selected for activation. These oocytes were placed in an activation solution containing 0.5 mM HEPES, 0.05 mM CaCl_2_-2H_2_O, 0.1 mM MgSO_4_·7H_2_O, and 0.01% polyvinyl alcohol (PVA) in 300 mM mannitol and activated using two direct current pulses of 120 V for 60 μs. Following activation, the oocytes were transferred to an IVC medium containing 7.5 mg/mL cytochalasin B and cultured for three hours at 38.5 °C and 5% CO_2_ to inhibit the expulsion of the second polar body. Subsequently, the oocytes were cultured in IVC medium (bicarbonate-buffered PZM-5 with 4 mg/mL bovine serum albumin [BSA]) at 38.5 °C, 5% CO_2_, and 100% humidity. The cleavage and blastocyst formation rates were recorded on days two and seven, respectively.

### 2.5. Determination of ROS and GSH Levels

Oocytes were collected after 44 or 68 h of IVM. The intracellular ROS levels were assessed by incubating the oocytes in ROS-stained droplets (PBS/PVA medium containing 10 µM H2DCFDA, Beyotime, Shanghai, China) for 30 min. Similarly, the GSH levels were measured by incubating the oocytes in GSH-stained droplets (PBS/PVA medium with 10 µM CMF2HC, Beyotime, Shanghai, China) for 30 min. Following staining, the oocytes were washed three times in PBS/PVA, and fluorescence images were captured using a fluorescence microscope (Nikon, Tokyo, Japan). The fluorescence intensities were analyzed using ImageJ version 8.0.2 software (NIH, Bethesda, MD, USA).

### 2.6. Determination of Mitochondrial Distribution

The mitochondrial distribution was evaluated by treating the oocytes with 500 nM Mito-Tracker Red CMXRos (Beyotime, Shanghai, China) for one hour at 38.5 °C in the dark, followed by three washes in PBS/PVA. Images were obtained under a fluorescence inverted microscope using the red fluorescence channel, and the fluorescence intensity was quantified using ImageJ version 8.0.2 software.

### 2.7. Mitochondrial Membrane Potential Assay

The mitochondrial membrane potential (MMP) was assessed by incubating oocytes in PBS/PVA medium containing 10 µM JC-1 (Beyotime, Shanghai, China) in the dark at 38.5 °C and 5% CO_2_ for 16 h. Fluorescence images were captured with a Nikon fluorescence microscope (Nikon, Tokyo, Japan), and the fluorescence intensity was analyzed with ImageJ version 8.0.2 software. The ratio of red to green fluorescence was calculated to determine the average MMP of the oocytes.

### 2.8. ATP Staining

Oocytes were washed in PBS/PVA and fixed in 3.7% paraformaldehyde for 30 min. They were then incubated with 500 nM BODIPY™ FL ATP dye (A12410; Thermo Fisher, Waltham, MA, USA) for one hour in the dark. Fluorescence images were captured using an inverted microscope, and the intensity was quantified using ImageJ version 8.0.2 software.

### 2.9. Intracellular Senescence-Associated β-Galactosidase (SA-β-Gal) Activity Assay

The intracellular SA-β-gal activity was measured using the Cellular Senescence Assay Kit (Spider-β gal, SG03, Dojindo, Kumamoto, Japan). Oocytes, with the ovarian mound removed, were incubated in 1 mL bafilomycin A1 working solution for one hour, followed by incubation in 1 mL SPiDER-βGAL working solution for 30 min, both in the dark at 38.5 °C and 5% CO_2_. Fluorescence images were taken using an inverted microscope, and the fluorescence intensity was analyzed with ImageJ software.

### 2.10. Annexin-V Staining

The oocyte apoptosis levels were evaluated using the Annexin V-FITC Apoptosis Detection Kit (Beyotime, Shanghai, China, C1062L), following the manufacturer’s instructions. Oocytes were incubated in annexin-V working solution (prepared by mixing 5 μL Annexin-V-FITC with 195 μL binding buffer) for 30 min in the dark at 37 °C. Fluorescence images were captured, and the early apoptosis rate was calculated to assess the impact of the treatment.

### 2.11. Immunofluorescent Staining

Oocytes were initially washed with PBS/PVA and then fixed in 3.7% paraformaldehyde for 30 min. They were permeabilized in PBS/PVA containing 0.1% Triton X-100 for 30 min, followed by a 1 h incubation in PBS/PVA with 3% BSA. Afterward, the oocytes were incubated overnight at 4 °C in the dark with the primary antibodies, including CHOP (1:300; Abcam, Cambridge, MA, USA: ab11419), PGC-1α (1:100; Abcam, Cambridge, MA, USA: ab72230), SIRT1 (1:100; Abcam, Cambridge, MA, USA: ab189494), and LC3B (1:200; Abcam, Cambridge, MA, USA: ab48394). The following day, the secondary antibodies were applied: anti-mouse IgG (1:500; CST, Danvers, MA, USA; 8890s) for CHOP staining, goat anti-rabbit antibody (1:500; Abcam, Cambridge, MA, USA; ab150077) for PGC-1α and LC3B, and anti-rabbit IgG (1:500; CST, Danvers, MA, USA; 8889s) for SIRT1 staining, with a 1 h incubation. After the antibody incubation, the oocytes were washed with PBS/PVA and stained with 10 µM Hoechst 33342 (Beyotime, Shanghai, China, C1025) for 8 min. The oocytes were then mounted onto slides and analyzed using fluorescence-inverted microscopy.

### 2.12. Total Cell Count of Blastocysts

To quantify the total cell count in the day-7 blastocysts, the blastocysts were collected, washed three to four times with PBS/PVA, and fixed in 3.7% paraformaldehyde for 30 min. They were subsequently stained with 10 μM Hoechst 33342 (Beyotime, Shanghai, China, C1025) for 8 min. After additional washing with PBS/PVA, the blastocysts were mounted on slides, and images of the nuclei were captured using a fluorescence microscope for analysis and cell counting.

### 2.13. RNA Extraction and qRT-PCR Assay

Oocytes from the fresh, aged, and DIOS-treated aged groups were collected. The total mRNA was extracted using the Dynabeads mRNA Direct Purification Kit (Invitrogen, Rochester, NY, USA) according to the manufacturer’s protocol. The extracted RNA was reverse transcribed into cDNA using the RevertAid First Strand cDNA Synthesis Kit (Thermo Fisher Scientific, Waltham, MA, USA). Gene expression analyses were performed with the KAPA SYBR FAST qPCR Master Mix (2X) Kit (Kapa Biosystems, Sigma-Aldrich, St. Louis, MO, USA, KK4601). Each PCR reaction contained 10 µL KAPA SYBR FAST qPCR Master Mix (2X), 0.4 µL ROX LOW, 8.8 µL cDNA template, and 0.4 µL each of the forward and reverse primers, bringing the total volume to 20 µL. qRT-PCR was carried out under the following conditions: initial denaturation at 95 °C for 3 min, followed by 40 cycles of denaturation at 95 °C for 3 s, annealing at 60 °C for 30 s, and extension at 72 °C for 20 s. The gene expression in the control and DIOS-treated groups was analyzed using QuantStudio^TM^ Design and Analysis version 1.5.2 Software. The quantitative results were calculated using the 2^−ΔΔCT^ method, with GAPDH serving as the internal reference gene. The analyzed genes and corresponding primers are listed in Table 1.

### 2.14. Statistical Analysis

The statistical results are presented as the mean ± standard deviation (SD). For comparisons between two groups, an independent samples *t*-test was used. For multiple group comparisons, a one-way ANOVA with the Tukey–Kramer post hoc tests was applied. All the statistical analyses were performed using SPSS version 22.0 (IBM Corporation, Chicago, IL, USA). Statistical significance was considered at * *p* < 0.05, ** *p* < 0.01, and *** *p* < 0.001.

## 3. Results

### 3.1. DIOS Supplementation Improves Cleavage Rate, Embryonic Developmental Competence, and Cell Number After Orphan Activation in Senescent Oocytes

**Figure 1:**
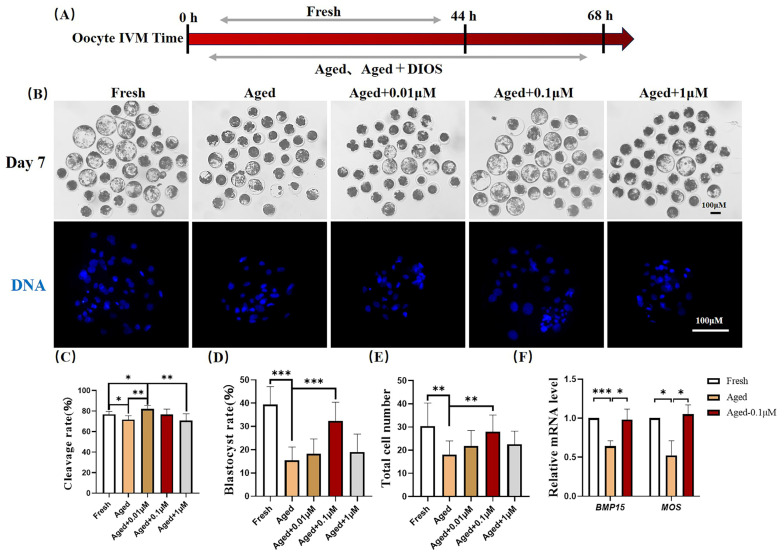
DIOS supplementation enhances the developmental capacity of aged oocytes during parthenogenetic activation. (**A**) IVM incubation times for the fresh, aged, and DIOS-treated aged groups. (**B**) Representative day-seven images of the embryonic development and Hoechst-stained blastocysts for the fresh, aged, and DIOS-treated aged oocytes. Scale bar = 100 μM. (**C**) Cleavage rates for fresh oocytes (N = 201), aged oocytes (N = 197), and aged oocytes treated with 0.01 μM (N = 210), 0.1 μM (N = 222), and 1 μM DIOS (N = 212). R = 5, * *p* < 0.05, ** *p* < 0.01. (**D**) Blastocyst rates for fresh oocytes (N = 289), aged oocytes (N = 267), and aged oocytes treated with 0.01 μM (N = 263), 0.1 μM (N = 280), and 1 μM DIOS (N = 284). R = 7, *** *p* < 0.001. (**E**) Blastocyst cell counts on day seven for fresh (N = 36), aged (N = 25), and DIOS-treated aged oocytes at 0.01 μM (N = 33), 0.1 μM (N = 43), and 1 μM (N = 25). R = 6, ** *p* < 0.01. (**F**) Differential expression of the *BMP15* and *MOS* genes in fresh, aged, and DIOS-treated aged oocytes. R = 3, * *p* < 0.05, *** *p* < 0.001.

The blastocyst formation rate on day seven and the corresponding blastocyst cell numbers for the fresh, aged, and DIOS-treated aged oocytes (with varying concentrations of DIOS: 0.01, 0.1, and 1 μM) are illustrated in Figure 1B. The blastocyst formation rates were as follows: 39.44 ± 7.66% for the fresh group, 15.53 ± 5.66% for the aged group, 18.29 ± 6.36% for the 0.01 μM DIOS-treated group, 32.39 ± 8.06% for the 0.1 μM DIOS-treated group, and 19.01 ± 7.72% for the 1 μM DIOS-treated group (Figure 1D). The corresponding blastocyst cell numbers were 30.44 ± 9.90, 18.00 ± 5.97, 21.79 ± 6.64, 28.00 ± 7.23, and 22.56 ± 5.65, respectively (Figure 1E). Additionally, the embryo cleavage rates were 76.78 ± 2.93% for the fresh group, 71.82 ± 3.70% for the aged group, 82.00 ± 3.34% for the 0.01 μM DIOS-treated group, 76.50 ± 5.45% for the 0.1 μM DIOS-treated group, and 70.72 ± 6.66% for the 1 μM DIOS-treated group (Figure 1C). The aging group showed a significant reduction in the embryo cleavage rates, blastocyst formation rates, and blastocyst cell numbers compared to the fresh group. Notably, 0.1 μM DIOS treatment significantly enhanced both the blastocyst formation rate and the blastocyst cell number in aged oocytes. In contrast, 0.01 μM DIOS significantly increased the embryo cleavage rate. These results suggest that DIOS plays a role in promoting the development of embryos derived from aged oocytes. Since the blastocyst rate and cell number more directly reflect the developmental capacity and quality of embryos, 0.1 μM DIOS was selected for the subsequent experiments. Furthermore, the expression levels of the *BMP15* (bone morphogenetic protein 15) and *MOS* (proto-oncogene, serine/threonine kinase) genes were significantly downregulated in the aged oocytes compared to the fresh group, with relative expression values of 0.64 ± 0.07 and 0.52 ± 0.19, respectively. However, DIOS treatment in the aged oocytes restored the expression of the *BMP15* (0.98 ± 0.14) and *MOS* (1.05 ± 0.12) genes (Figure 1F).

### 3.2. DIOS Supplementation Enhances the Antioxidant Capacity of Senescent Oocytes

**Figure 2:**
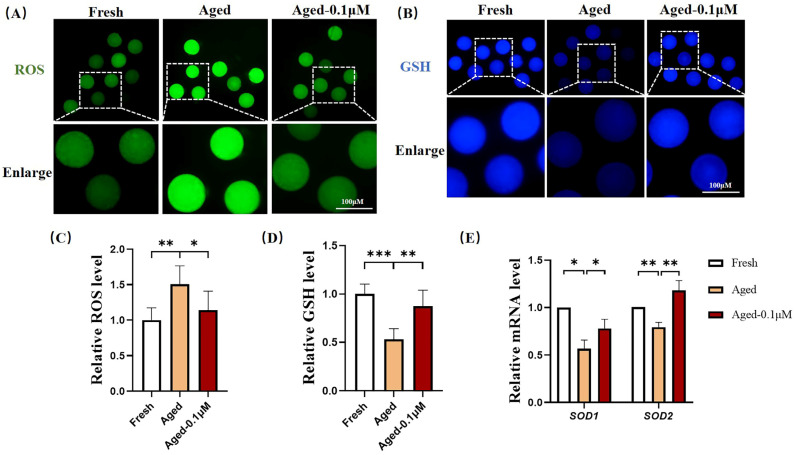
Antioxidant effects of DIOS on aged oocytes. (**A**) Representative ROS staining images of oocytes from the fresh, aged, and DIOS-treated aged groups. Scale bar = 100 μM. (**B**) Representative GSH staining images of oocytes from the fresh, aged, and DIOS-treated aged groups. Scale bar = 100 μM. (**C**) Relative ROS fluorescence intensity in fresh (N = 40), aged (N = 43), and DIOS-treated aged oocytes (N = 37). R = 4, * *p* < 0.05, ** *p* < 0.01. (**D**) Relative GSH fluorescence intensity in fresh (N = 31), aged (N = 33), and DIOS-treated aged oocytes (N = 31). R = 3, ** *p* < 0.01, *** *p* < 0.001. (**E**) Differential expression of antioxidant genes in fresh, aged, and DIOS-treated aged oocytes. R = 3, * *p* < 0.05, ** *p* < 0.01.

To assess the antioxidant effects of DIOS on aged oocytes, the ROS and GSH levels were evaluated (Figure 2A,B). The results showed that the ROS levels were significantly higher in the aged oocytes compared to the fresh oocytes (1.51 ± 0.26). However, DIOS treatment in the aged oocytes led to a significant reduction in the ROS levels (1.14 ± 0.27) (Figure 2C). Regarding the GSH levels, the aged oocytes displayed a notable decrease (0.53 ± 0.11) compared to the fresh oocytes, while the DIOS-treated aged oocytes exhibited a substantial increase in the GSH levels (0.87 ± 0.17) (Figure 2D). Additionally, the expression of the antioxidant genes superoxide dismutase 1 (*SOD1*) and superoxide dismutase 2 (*SOD2*) was significantly lower in the aged oocytes (0.57 ± 0.09 and 0.79 ± 0.05, respectively) compared to the fresh oocytes. In contrast, DIOS treatment significantly upregulated these genes’ expression to 0.78 ± 0.10 for SOD1 and 1.18 ± 0.10 for SOD2 (Figure 2E).

### 3.3. DIOS Supplementation Improves Mitochondrial Function in Senescent Oocytes

**Figure 3:**
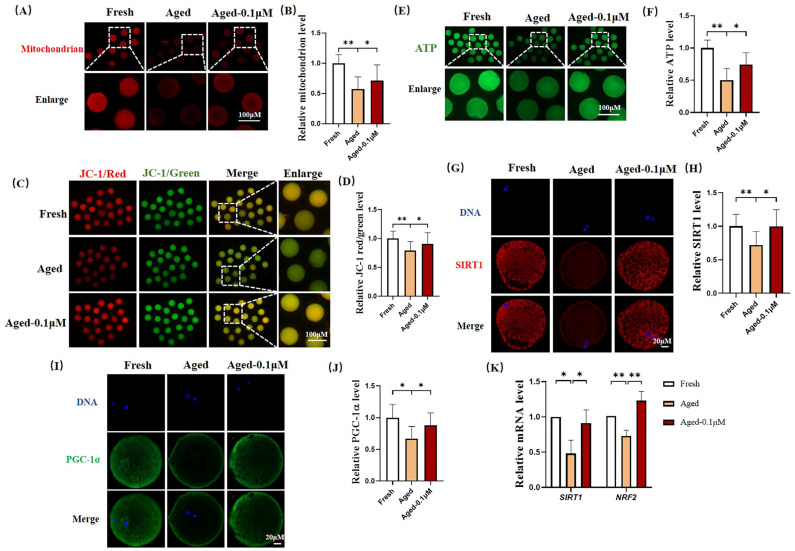
Effects of DIOS on mitochondrial function in aged oocytes. (**A**) Representative images of the mitochondrial abundance staining in the fresh, aged, and DIOS-treated aged oocyte groups. Scale bar = 100 μM. (**B**) Relative mitochondrial abundance in the fresh (N = 29), aged (N = 32), and DIOS-treated aged oocyte groups (N = 31). R = 3, * *p* < 0.05, ** *p* < 0.01. (**C**) Representative JC-1 staining images in the fresh, aged, and DIOS-treated aged oocyte groups. Scale bar = 100 μM. (**D**) Relative JC-1 red/green fluorescence intensity in the fresh (N = 61), aged (N = 59), and DIOS-treated aged oocyte groups (N = 65). R = 4, * *p* < 0.05, ** *p* < 0.01. (**E**) Representative ATP staining images in the fresh, aged, and DIOS-treated aged oocyte groups. Scale bar = 100 μM. (**F**) Relative ATP levels in the fresh (N = 36), aged (N = 34), and DIOS-treated aged oocyte groups (N = 35). R = 3, * *p* < 0.05, ** *p* < 0.01. (**G**) Representative SIRT1 staining images in the fresh, aged, and DIOS-treated aged oocyte groups. Scale bar = 20 μM. (**H**) Relative SIRT1 fluorescence intensity in the fresh (N = 45), aged (N = 41), and DIOS-treated aged oocyte groups (N = 46). R = 3, * *p* < 0.05, ** *p* < 0.01. (**I**) Representative PGC-1α staining images in the fresh, aged, and DIOS-treated aged oocyte groups. Scale bar = 20 μM. (**J**) Relative PGC-1α fluorescence intensity in the fresh (N = 59), aged (N = 58), and DIOS-treated aged oocyte groups (N = 57). R = 3, * *p* < 0.05. (**K**) Differential gene expression in fresh, aged, and DIOS-treated aged oocytes. R = 3, * *p* < 0.05, ** *p* < 0.01.

Oxidative stress is closely linked to mitochondrial function. To examine the effects of DIOS on mitochondrial function in aged oocytes, the mitochondrial distribution, membrane potential, and ATP levels were assessed (Figure 3A,C,E). A significant reduction in the mitochondrial fluorescence intensity was observed in the aged oocytes (0.57 ± 0.20) compared to the fresh oocytes, while DIOS treatment enhanced the mitochondrial fluorescence intensity in the aged oocytes (0.72 ± 0.26) (Figure 3B). The JC-1 red/green fluorescence ratio, a marker of the mitochondrial membrane potential, was notably lower in the aged oocytes (0.80 ± 0.15) than in the fresh oocytes, but DIOS treatment improved this ratio in the aged oocytes (0.91 ± 0.20) (Figure 3D). The ATP fluorescence intensity, reflecting intracellular ATP production, was also significantly reduced in the aged oocytes (0.50 ± 0.18) compared to the fresh oocytes. In contrast, the DIOS-treated aged oocytes exhibited a significant increase in the ATP levels (0.74 ± 0.19) (Figure 3F). Moreover, the fluorescence intensity of the mitochondrial proteins critical for growth and development, such as SIRT1 and PGC-1α, was significantly reduced in the aged oocytes (0.72 ± 0.20 and 0.67 ± 0.19, respectively) compared to the fresh oocytes. DIOS treatment, however, led to a substantial increase in these protein levels in the aged oocytes (1.00 ± 0.25 and 0.88 ± 0.20) (Figure 3G–J). Finally, the expression levels of two mitochondria-related genes, silent mating type information regulation 2 homolog-1 (*SIRT1*) and nuclear respiratory factor 2 (*NRF2*), were assessed. Compared to the fresh group, the aged group showed significant downregulation of *SIRT1* and *NRF2* expression (0.48 ± 0.19 and 0.73 ± 0.08, respectively). However, DIOS treatment notably upregulated the expression of these genes in the aged oocytes (0.91 ± 0.19 and 1.23 ± 0.13) (Figure 3K).

### 3.4. DIOS Reduces Apoptosis Levels by Mitigating Cellular Senescence

**Figure 4:**
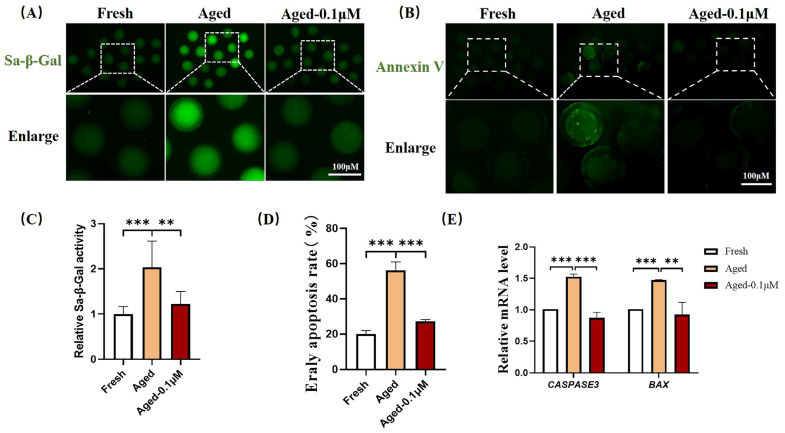
Effect of DIOS on senescence and early apoptosis in aged oocytes. (**A**) Representative SA-β-Gal staining images in fresh, aged, and DIOS-treated aged oocytes. Scale bar = 100 μm. (**B**) Representative annexin-V staining images in fresh, aged, and DIOS-treated aged oocytes. Scale bar = 100 μm. (**C**) Relative SA-β-Gal activity in fresh (N = 53), aged (N = 50), and DIOS-treated aged oocytes (N = 63). R = 3, ** *p* < 0.01, *** *p* < 0.001. (**D**) Early apoptosis rates in fresh (N = 85), aged (N = 77), and DIOS-treated aged oocytes (N = 77). R = 4, *** *p* < 0.001. (**E**) Expression levels of apoptosis-related genes in fresh, aged, and DIOS-treated aged oocytes. R = 3, ** *p* < 0.01, *** *p* < 0.001.

To assess the impact of DIOS on oocyte senescence, the activity of the senescence marker SA-β-Gal was evaluated. As shown in Figure 4A,C, the SA-β-Gal activity was significantly elevated in the aging group (2.04 ± 0.58) compared to the fresh group, while DIOS treatment notably reduced the SA-β-Gal activity in the aged oocytes (1.22 ± 0.28). These results suggest that DIOS effectively mitigates the rate of senescence in aged oocytes.

The apoptosis, a key hallmark of degeneration in aged oocytes [25], was also investigated. As indicated in Figure 4B, strong annexin-V fluorescence signals were observed in the aging group, while such signals were nearly absent in both the fresh and DIOS-treated aged oocytes. The proportion of oocytes exhibiting green fluorescence was significantly higher in the aged group (56.25 ± 4.79%) compared to the fresh group (20.05 ± 1.90%), but it was significantly reduced in the DIOS-treated aged oocytes (27.35 ± 0.87%) (Figure 4D). Furthermore, the expression levels of the apoptosis-related genes *CASPASE3* and *BAX* were measured. Both *CASPASE3* (1.53 ± 0.04) and *BAX* (1.47 ± 0.01) were significantly upregulated in the aged oocytes compared to the fresh oocytes, while DIOS treatment resulted in a marked reduction in the expression of these genes (CASPASE3: 0.87 ± 0.09, BAX: 0.92 ± 0.19) (Figure 4E).

### 3.5. DIOS Supplementation Alleviates Endoplasmic Reticulum Stress Levels in Senescent Oocytes

**Figure 5:**
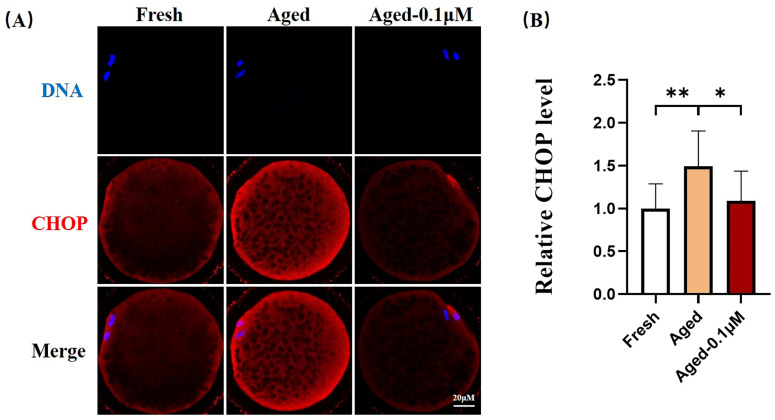
DIOS regulates endoplasmic reticulum homeostasis in aged oocytes. (**A**) Immunostaining of the CHOP protein in the fresh, aged, and DIOS-treated aged oocyte groups. Scale bar = 20 μM. (**B**) Relative CHOP fluorescence intensity in fresh (N = 45), aged (N = 41), and DIOS-treated aged oocytes (N = 39). R = 3, * *p* < 0.05, ** *p* < 0.01.

The ERS levels in the oocytes were assessed by immunofluorescence staining of CHOP, a marker of ER stress (Figure 5A). The results indicated a significant increase in ERS in the aged oocytes relative to the fresh group (1.49 ± 0.41), whereas DIOS treatment notably reduced the ERS levels in the aged oocytes (1.09 ± 0.35) (Figure 5B).

### 3.6. DIOS Supplementation Inhibits Autophagy in Senescent Oocytes

**Figure 6:**
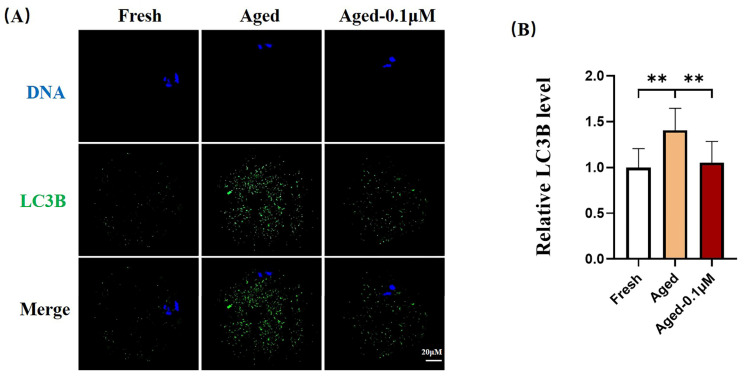
DIOS impacts autophagy in senescent oocytes. (**A**) Immunofluorescence staining of LC3B in the fresh, aged, and DIOS-treated aged oocyte groups. Scale bar = 20 μM. (**B**) Relative LC3B fluorescence intensity in fresh (N = 67), aged (N = 65), and DIOS-treated aged oocytes (N = 67). R = 3, ** *p* < 0.05.

To examine the effect of DIOS on autophagy in senescent oocytes, the LC3B expression was quantified. Compared to the fresh group, the LC3B fluorescence intensity was significantly higher in the aged oocytes (1.41 ± 0.24) but substantially decreased in the DIOS-treated aged oocytes (1.05 ± 0.23) (Figure 6A,B).

## 4. Discussion

This study investigates the effect of DIOS on porcine oocyte aging in vitro. High-quality oocytes are essential for successful fertilization and subsequent embryo development [26]. Oocyte quality declines in a time-dependent manner with prolonged IVM [27]. Numerous studies indicate that the optimal maturation time for porcine oocytes is 42–46 h [28,29]. Therefore, a 44 h maturation period was designated as the fresh group, while oocytes cultured for 68 h were classified as the aging group. DIOS at various concentrations was added to the culture for 68 h in the treatment group to assess its impact on oocyte aging.

The results showed that DIOS improved the quality of aged oocytes and the developmental potential of embryos derived from these oocytes, as evidenced by the enhanced blastocyst formation, cleavage rates, and blastocyst cell numbers. Compared to the fresh group, the blastocyst rate, cleavage rate, and blastocyst cell number were significantly lower in the aging group, but DIOS treatment mitigated these effects. Notably, 0.1 μM DIOS improved both the blastocyst rate and the cell number, while 0.01 μM DIOS enhanced the cleavage rate in aging oocytes. These findings suggest that DIOS’s impact on embryonic development may extend beyond promoting cleavage. In addition, high concentrations of DIOS at 1 μM may inhibit its growth and development potential. Previous studies have shown that high concentrations of DIOS have toxic effects on cells and reduce cell activity [30]. Therefore, the inhibition of the growth and development of aged oocytes by a 1 μM high concentration of DIOS may be due to its toxic effect and reduced activity. Additionally, changes in the mRNA expression of key functional genes, such as *BMP15* and *MOS*, were observed. *BMP15*, a member of the TGF-β superfamily, is critical for oocyte maturation and follicular development [31]. Its expression is elevated in high-quality embryos [32] and decreases with age [33]. Similarly, *MOS*, a vital gene in oocyte maturation [34], regulates meiosis in mammalian oocytes. The MOS protein kinase activates the mitogen-activated protein kinase (MAPK) [35], which stabilizes the maturation-promoting factor (MPF) through the MOS/MAPK pathway during oocyte maturation [36]. Consistent with previous studies, the reduced oocyte quality in the aging group correlated with decreased *BMP15* and *MOS* expression, impairing oocyte maturation and embryonic development. However, DIOS treatment enhanced the BMP15 and MOS expression, suggesting that DIOS supports oocyte maturation and enhances both oocyte quality and embryonic development.

Aging oocytes exhibit decreased quality post-ovulation, increasing the risk of miscarriage and congenital malformations, primarily due to oxidative stress from ROS accumulation [37]. Our findings indicate that ROS accumulation in aging oocytes induces oxidative stress, leading to a reduction in intracellular antioxidants, such as GSH, and downregulation of antioxidant genes (*SOD1*, *SOD2*), thereby weakening the oocytes’ antioxidant defenses. DIOS treatment effectively reduced the ROS levels, increased the GSH, and upregulated the antioxidant gene expression, suggesting that DIOS alleviates ROS-induced oxidative stress in aging oocytes.

Mitochondria are the primary energy source for oocytes, being essential for oocyte maturation, fertilization, and embryonic development by generating ATP to support various cellular functions [38,39,40]. The majority of ATP required for pre-implantation embryonic development originates from the mitochondria of MII-stage oocytes [41], highlighting the importance of mitochondrial function in oocyte development. However, aging oocytes exhibit mitochondrial dysfunction, characterized by a decrease in the mitochondrial membrane potential and disruption to the mitochondrial distribution [42]. SIRT1 is a key player in the mitochondrial biosynthesis signaling pathway [43], while PGC-1α is a transcriptional regulator that controls mitochondrial biogenesis, metabolic processes, and oxidative stress [44]. SIRT1 and PGC-1α interact closely, with SIRT1 acting as an upstream regulator, deacetylating PGC-1α to enhance its activity, thus promoting mitochondrial biogenesis and preserving mitochondrial function [45,46,47]. In this study, aging oocytes displayed a marked decline in the mitochondrial membrane potential, disrupted mitochondrial distribution, and reduced expression of SIRT1, PGC-1α, and mitochondria-related genes such as *SIRT1* and *NRF2*, leading to impaired ATP production. DIOS treatment, however, restored these parameters, improving mitochondrial function and ATP production, ultimately enhancing the quality of aging oocytes.

SA-β-Gal, a well-established marker of cellular senescence, exhibits increased activity as cells undergo senescence [48]. Assessing the SA-β-Gal activity in oocytes provides a reliable indicator of oocyte aging. Cellular senescence is closely associated with apoptosis, characterized by elevated levels of apoptosis-related proteins and increased apoptosis rates [49]. Our analysis revealed a significant increase in SA-β-Gal activity in aging oocytes, signifying pronounced senescence. This was accompanied by a higher rate of early apoptosis and upregulation of pro-apoptotic genes (*CASPASE3*, *BAX*). In contrast, DIOS treatment significantly reduced both the SA-β-Gal activity and the early apoptosis rates, along with the expression of these pro-apoptotic genes. These findings suggest that DIOS may mitigate apoptosis by delaying oocyte senescence.

Previous studies have demonstrated that ERS impairs the developmental competence of bovine oocytes, with ERS inhibitors enhancing the maturation rates, reducing the apoptosis and ROS levels, and improving the blastocyst formation during early embryonic development [50]. CHOP, a critical marker of ERS, is upregulated under cellular stress conditions [51]. In this study, aging oocytes exhibited significantly elevated CHOP expression, indicating severe ERS. DIOS treatment, however, reversed this effect, suggesting that DIOS may alleviate senescence-induced ERS, thereby enhancing the developmental potential of aging oocytes. Furthermore, the autophagy levels in aged oocytes were evaluated. Autophagy, a form of programmed cell death, can play a dual role: moderate autophagy supports cellular maintenance and homeostasis, while excessive autophagy can lead to cell death [52]. LC3B, a key regulator of autophagy, serves as a critical marker in this process [53]. Our results showed that the LC3B levels were significantly elevated in senescent oocytes, indicating excessive autophagy. DIOS supplementation reduced these elevated autophagy levels, suggesting that DIOS helps stabilize cellular conditions and preserve the quality of senescent oocytes by inhibiting excessive autophagy.

## 5. Conclusions

**Figure 7:**
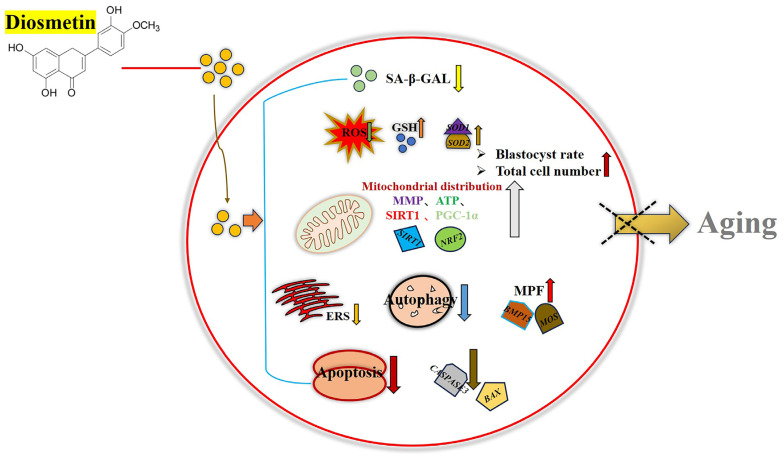
Schematic overview of DIOS’s effects on oocyte aging. DIOS treatment reduced the intracellular ROS, SA-β-Gal, ERS, apoptosis, and autophagy in senescent oocytes while enhancing the intracellular GSH levels, mitochondrial function, and expression of key genes. These combined effects contributed to the mitigation of oocyte aging, improving the oocyte quality and boosting the developmental potential of early embryos derived from aged oocytes.

This study highlights the potential of DIOS, an antioxidant, in delaying oocyte aging in vitro. The results demonstrate that DIOS effectively mitigates oocyte aging by reducing apoptosis, oxidative stress, endoplasmic reticulum stress, and autophagy. Additionally, DIOS enhances mitochondrial function, which increases ATP production and upregulates maturation-inducing factors, ultimately improving the quality of aged oocytes. These improvements result in a higher blastocyst formation rate and an increase in the number of blastocyst cells in aged oocytes (Figure 7).

## Figures and Tables

**Table 1:** Primer sequences used for real-time PCR.

Genes	Primer Sequences (5′-3′)	Base
*GAPDH*	F: TTCCACGGCACAGTCAAG R: ATACTCAGCACCAGCATCG	18 19
*BMP15*	F: CCTCCATCCTTTCCAAGTCA R: GTGTAGTACCCGAGGGCAGA	20 20
*MOS*	F: TGGGAAGAAACTGGAGGACA R: TTCGGGTCAGCCCAGTTCA	20 19
*SOD1*	F: CAAAGGATCAAGAGAGGCACG R: CGAGAGGGCGATCACAGAAT	21 20
*SOD2*	F: TTCTGGACAAATCTGAGCCCTAACG R: CGACGGATACAGCGGTCAACTTC	25 23
*NRF2*	F: AGCGGATTGCTCGTAGACAG R: TTCAGTCGCTTCACGTCGG	20 19
*SIRT1*	F: ACAGGTTGCAGGAATCCAGAG R: TAGGACATCGAGGAACCACCT	21 21
*CASP3*	F: AGAATTGGACTGTGGGATTGAGACG R: GCCAGGAATAGTAACCAGGTGCTG	25 24
*BAX*	F: GGACTTCCTTCGAGATCGGC R: GCGTCCCAAAGTAGGAGAGG	20 20

Abbreviation: PCR, polymerase chain reaction.

## Data Availability

The data supporting the findings of this study are available from the corresponding author upon reasonable request.
